# Special Issue “Osteosarcomas: Treatment Strategies”

**DOI:** 10.3390/ph16091233

**Published:** 2023-08-31

**Authors:** See-Hyoung Park

**Affiliations:** Department of Biological and Chemical Engineering, Hongik University, Sejong 30016, Republic of Korea; shpark74@hongik.ac.kr

This Special Issue, titled “Osteosarcomas: Treatment Strategies”, aims to overview the recent and future research trends related to the treatment of osteosarcoma. We thus invited both original research articles and review papers that shed light on the challenges in developing therapeutic strategies for the treatment of osteosarcoma. More especially, the topics of interest include drug development, drug repositioning, the selective optimization of lead compounds, and drug combinations. Osteosarcoma, also known as bone tumors, generally affects children and adolescents. According to recent cancer statistics, osteosarcoma was ranked as having the lowest five-year relative survival rate among the most common childhood and adolescent cancers in the United Sates [1,2]. Since modern therapeutic methods have developed rapidly, the outcome for patients with osteosarcoma has improved. The typical treatments for osteosarcoma include surgery and intensive chemotherapy [3,4]. However, with respect to recurrent and metastatic osteosarcoma, existing treatments, including surgery and chemotherapy, exhibit a limited therapeutic effect. Therefore, it is necessary to develop new therapeutic strategies to treat osteosarcoma. Our call for papers for this Special Issue received seven research articles and one review paper.

In the first article, Hsieh et al. from Taiwan demonstrated the anti-metastatic activity of Timosaponin AIII (TSAIII), a steroidal saponin [5]. They showed that TSAIII inhibited the distribution of cytoskeletal F-actin and cytoskeletal-related proteins, which contributed to the reduction in osteosarcoma cell migration and invasion without reducing cell growth or apoptosis. They also confirmed that synergistic inhibitory effects on osteosarcoma cell migration and invasion was induced through the cotreatment of TSAIII with V3 integrin inhibitor (Cyclo(RGDyK) peptide) or focal adhesion kinase inhibitor (PF-573228). Finally, they examined that TSAIII strongly suppressed the pulmonary metastasis of osteosarcoma cells in vivo. These discoveries suggested that TSAIII could be a potential method of supplemental therapy in human osteosarcoma metastasis.

In the second article, Danieau et al. from France and the UK reported that the β-Catenin inhibitor (ICG-001) decreased the proliferation, but, surprisingly, increased the migration rate of, osteosarcoma cells [6]. According to these authors, ICG-001 treatment inhibited proliferation in osteosarcoma cells by blocking the β-catenin-dependent transcription and arresting the cell cycle in the G0/G1 phase. Interestingly, ICG-001 did increase the migration rate in osteosarcoma cells, which was confirmed through metastatic dissemination to the lungs in an in vivo mouse model. The authors explained that this discrepancy between proliferation and migration might be caused by differentiations in the treatment dose and time. They suggested that the unexpected pro-metastatic role of ICG-001 is of potential interest in the careful consideration of treatments for osteosarcoma.

In the third article, Lu et al. from Taiwan showed that the curcumin analog (GO-Y078) triggered both intrinsic and extrinsic apoptotic pathways in osteosarcoma cells through the activation of JNK and p38 MAPK [7]. They showed that GO-Y078 treatment decreased the viability of osteosarcoma cells and induced apoptosis with an increase in SubG1 population. In addition, they demonstrated that GO-Y078 treatment significantly activated caspase 3, caspase 8 (an initiator of extrinsic apoptosis), and caspase 9 (an initiator of intrinsic apoptosis) but downregulated the expression of anti-apoptotic IAP-1 and XIAP. They provided evidence that JNK and p38 MAPK were involved in the regulation of proliferation and apoptosis by GO-Y078 in osteosarcoma cells. This study contributes to our understanding of the more detailed mechanisms of the apoptotic activity of GO-Y078 in osteosarcoma cells.

In the fourth article, Aziz et al. from Malaysia and Pakistan examined the anti-metastatic and anti-angiogenic activity of another curcumin analog (DK1) in osteosarcoma cells [8]. Interestingly, compared to the anti-apoptotic activity of GO-Y078 from the third article by Lu et al., DK1 displayed anti-metastatic and anti-angiogenic activity in osteosarcoma cells. Through an analysis of the microarray gene expression and proteome profile, DK1 was proven to play a pivotal role in controlling the signaling pathways responsible for metastasis and angiogenesis, such as PI3K/Akt and NF-κB, in osteosarcoma cells. Since metastasis is one of the challenging aspects in osteosarcoma, their results implied that DK1 could be considered as an anti-metastasis compound for treating the cancer.

In the fifth article, Koons et al. from the USA and Israel refined a phenotypic screening process to identify potential drugs that can impede the metastatic progression of osteosarcoma cells [9]. They validated a previously developed three-dimensional cell culture model for simulating the metastatic growth of metastatic osteosarcoma cells. Then, they confirmed the reliability of their assay system by investigating the effects of two kinds of anti-cancer drugs (regorafenib and saracatinib) and demonstrated an alignment between their assay results and human clinical trials. Additionally, they investigated the possibility of a veterinary-approved anti-atopic drug (oclacitinib) as a potential candidate for treating human osteosarcoma but unfortunately found no substantiation for oclacitinib’s effectiveness in the anti-metastasis of osteosarcoma cells. These findings underscore the fact that, unlike traditional anti-cancer drug discovery methods that primarily assess the cytotoxic effects on actively dividing cells, their approach focuses on inhibiting the spread of osteosarcoma cells.

In the sixth article, Broqueza et al. from Canada and the USA reported the anti-cancer activity of a 177 Lutetium labeled human antibody in IGF2R in a SCID mouse model bearing canine-derived osteosarcoma [10]. They developed a new type of antibody called IF3, which specifically binds to the IGF2R receptor in both human and canine osteosarcoma. The radioimmunotherapy using 177 Lutetium labeled IF3 was successful in significantly inhibiting tumor growth in mice with canine osteosarcoma, although there were some indications of spleen-related side effects. They emphasized that it is crucial to assess the potential toxicity of this treatment in healthy dogs before considering clinical trials for treating osteosarcoma.

In the seventh article, Luca et al. from Italy examined the diverse impacts of resveratrol in osteosarcoma cells [11]. They observed the inhibition of cell growth and induction of apoptosis via the regulation of Akt and caspase 3 through resveratrol treatment in osteosarcoma cells. They also showed that resveratrol inhibited cell migration, which was linked to an epigenetic reduction in proinflammatory IL-6 and IL-8. Interestingly, they found that resveratrol promoted osteoblastic differentiation through the significant upregulation of osteoblastic differentiation genes. Furthermore, the combined treatment of resveratrol with conventional chemotherapeutic agents, such as doxorubicin and cisplatin, intensified their cytotoxic activity in osteosarcoma cells. They suggested that, after further investigations, resveratrol might be one of the potential therapeutic supplements for treating osteosarcoma.

In the eighth review paper, Argenziano et al. from Italy reviewed the recent papers regarding novel therapeutic approaches for treating osteosarcoma [12]. They provided an overview of research addressing the possibilities of proteasome inhibitors, endocannabinoid/endovanilloid receptor agonists, immune system activators, antibodies, and iron chelators in the treatment of osteosarcoma. In addition, they introduced Sorafenib (a tyrosine kinase inhibitor), Eribulin (a microtubule inhibitor), Glembatumumab vedotin (an antibody–drug conjugate), and antibodies against disialoganglioside as emerging anti-cancer drugs in treating osteosarcoma. They concluded that these approaches have the potential to improve outcomes for treating osteosarcoma by addressing the current challenges, such as resistance to chemotherapy and unexpected side effects.

In summary, this Special Issue highlighted the recent challenges for treating osteosarcoma. Six articles investigated the anti-cancer activity of small chemical entities, one article showed a novel screening system using three-dimensional cell culture, and one review paper summarized the recent novel therapeutic options for treating osteosarcoma. Although there are still several unexplained questions identified in these studies, we hope that researchers around the world will gain inspiration from this Special Issue, and that it may lead to the development of new therapeutic approaches for osteosarcoma. We are sincerely grateful to the authors and reviewers for their contributions to this Special Issue.
